# A Text-Book of Materia Medica, Therapeutics, and Pharmacology

**Published:** 1897-12

**Authors:** 


**A Text-Book of Materia Medica, Therapeutics, and Pharmacology.** By GEORGE FRANK BUTLER, M.D. Pp. 858. London: The Rebman Publishing Co. (Ltd.). Philadelphia: W. B. Saunders. 1897.

Of the making of American text-books of Materia Medica there seems to be no end. Perhaps the multiplicity of American schools partly accounts for this; although this particular manual is used outside the author's own medical school at Chicago. A Montreal student told us it was the accepted text-book there.

The arrangement of remedies is based upon therapeutic affinities—one we believe to be at present unpractical, even if theoretically philosophical: the alphabetical arrangement of substances we consider the most convenient. There is an excellent short chapter on weights and measures, and a much longer one on prescriptions, which latter, however, is by no means free from errors in its Latinity. Upon the whole we would commend the book as being reasonable, careful, and concise. It is attractively sent out by the publishers as one of their "Blue Cover Series."

